# Intestinal Microbiota Structure of Xichuan Black-Bone Chickens and Preliminary Evaluation of a Probiotic-Based Fecal Microbiota Substitute

**DOI:** 10.3390/microorganisms14091966

**Published:** 2026-09-05

**Authors:** Lin Yuan, Chen Zhang, Wanli Li, Jinfan Yi, Chaoqi Liu, Wei Jin, Guoxi Li, Bingxun Wang, Shengli Li, Haoyu Wang

**Affiliations:** 1 Henan Key Laboratory of Animal Breeding and Nutritional Regulation, Institute of Animal Husbandry, Henan Academy of Agricultural Sciences, Zhengzhou 450002, China; yuanlin315@126.com (L.Y.); zhangchen19940205@163.com (C.Z.); rhettbutler@126.com (W.J.); wbx13298185299@126.com (B.W.); aabb26@126.com (S.L.); xumusuowanghaoyu@163.com (H.W.); 2 The Shennong Laboratory, Zhengzhou 450002, China; yijinfan0331@163.com (J.Y.); liguoxi0914@126.com (G.L.); 3 College of Animal Science and Technology, Henan Agricultural University, Zhengzhou 450046, China; cql2021@126.com

**Keywords:** Xichuan black-bone chickens, intestinal microbiota, metagenomic sequencing, fecal microbiota transplantation, probiotic strain

## Abstract

In this study, we aimed to explore the intestinal microbiota structure of Xichuan black-bone chickens (XBCs) and evaluate the effect of fecal microbiota substitute transplantation (FMST) by comparing it with traditional fecal microbiota transplantation (FMT). Metagenomic sequencing was used to analyze the microbiota composition and diversity of different intestinal segments (duodenum, jejunum, ileum, cecum, and rectum) of adult XBCs. Probiotic strains were subsequently isolated and screened from the cecal contents under anaerobic conditions to prepare FMST preparations. In total, 90 1-day-old XBCs were randomly divided into the FMT group, FMST group and control group (CK) for the transplantation experiment. The results revealed that the cecum had the highest species richness among all intestinal segments, with a mean species number of 7593.2. Three probiotic strains, namely, *Lactobacillus crispatus*, *Weissella paramesenteroides* and *Bacillus amyloliquefaciens*, were successfully screened and identified. In the transplantation experiment, the FMT group exhibited optimal α diversity of the cecal microbiota, while compared with the FMT group, the FMST group had significantly reduced expression of pro-inflammatory cytokines (IL-6, TNF-α, and IL-1β) and increased expression of intestinal tight junction proteins (claudin-1 and ZO-1). In conclusion, the cecum of XBCs has the most abundant microbial resources. Under the short-term intervention model tested herein, the custom FMST formulation delivers superior intestinal protective effects and shows promising potential as a standardized alternative to conventional FMT, which is highly important for the standardized application of fecal microbiota transplantation in poultry production.

## 1. Introduction

Xichuan black-bone chickens (XBCs), a native chicken breed in China, are characterized by “five blacks” and green-shell egg production [1]. The breed has the advantages of strong disease resistance, a high slaughter rate, and medicinal and health care functions, integrating meat, egg and medicinal uses [2]. The intestinal microbiota, the community of microorganisms in the host intestine, plays important roles in nutrient absorption, immune system development and disease resistance [3]. Exploring the intestinal microbiota structure of XBCs is crucial for understanding its genetic characteristics in terms of high disease resistance.

Fecal microbiota transplantation (FMT), which transfers the fecal microbiota of healthy donors to recipients with intestinal disorders, has been applied in poultry breeding to promote intestinal development [4], improve laying performance [5] and reduce Salmonella infection [6]. However, FMT has potential risks, such as pathogen transmission, low recipient acceptance and non-standardized treatment protocols [7,8,9]. Therefore, it is necessary to screen and optimize the fecal microbiota to prepare safe and effective substitute preparations. In this study, the intestinal microbiota structure of XBCs was analyzed, probiotic strains were screened to prepare FMST preparations, and the application effect of these strains was verified through transplantation experiments, providing a theoretical basis for the standardized application of FMT in the poultry industry.

## 2. Materials and Methods

### 2.1. Experimental Animals

Five healthy adult male XBCs were selected for intestinal microbiota structure analysis. For the transplantation experiment, 90 1-day-old XBCs with a consistent genetic background and an initial average body weight of 28.6 ± 1.5 g were obtained from the breeding base in Shangcai County, Zhumadian City, China.

### 2.2. Sample Collection and Grouping

The chickens were fasted for 12 h with free access to water before sampling. They were anesthetized by intravenous injection of 25 mg/kg of thiopental sodium (Takara, Dalian, China). After a few minutes, the broilers fell, they experienced general weakness, reaction disappeared, and they were euthanized by jugular vein bloodletting. The intestinal segments, including the duodenum (10–15 cm), jejunum (30–40 cm), ileum (15–20 cm), cecum (20–30 cm, a pair) and rectum (5–10 cm), were separated on a sterile operating table. The contents of each intestinal segment were squeezed into sterile cryopreservation tubes, labeled with the chicken number, intestinal segment name and collection time, and stored in liquid nitrogen for subsequent metagenomic analysis. The samples were divided into five groups according to intestinal segments: group A (duodenum), group B (jejunum), group C (ileum), group D (cecum) and group E (rectum), with five replicates in each group.

For the transplantation experiment, the chickens were randomly divided into three groups with 30 chickens in each group (half male and half female) and three replicates of 10 chickens each: the FMT group (oral administration of the fecal microbiota solution), the FMST group (oral administration of the fecal microbiota substitute preparation) and the CK group (oral administration of sterile normal saline).

### 2.3. DNA Extraction and Metagenomic Sequencing

The intestinal content samples were thawed on ice, and 0.2 g of each sample was homogenized with 0.8 mL of sterile PBS buffer. The homogenate was centrifuged twice at 8000 r/min at 4 °C for 5 min to remove impurities. DNA was extracted using a commercial kit (Beyotime, Shanghai, China), and its purity (OD260/OD280: 1.8–2.0; OD260/OD230: 2.0–2.5), integrity and concentration (≥50 ng/μL; total mass ≥1 μg) were determined.

Qualified DNA samples were fragmented into 300–500 bp fragments by ultrasonic disruption. After end repair, A-tailing and adapter ligation, PCR amplification was performed, and the products were subsequently purified to construct sequencing libraries. These libraries were sequenced using PE150 paired-end reads on the Illumina NovaSeq 6000 (Illumina, San Diego, CA, USA) platform for metagenomic libraries. The raw data were filtered using Trimmomatic (v0.39) software to obtain clean data, and host genome sequences were removed by alignment with the chicken reference genome (Gallus gallus-6.0) using Bowtie2 (v2.4.5) software. MEGAHIT was used for primary metagenomic assembly, IDBA-UD was applied to a small subset of samples for comparative testing, and gene prediction and annotation were performed using Prodigal (v2.6.3) software and public databases (NR, KEGG, COG, and Swiss-Prot).

### 2.4. Isolation, Purification and Identification of Probiotic Strains

FMT donor: Cecal digesta for preparing raw FMT inoculum was pooled from five clinically healthy adult XBC (60–90 days old, 1.5–2.0 kg) donors. One cecal sample was collected per individual bird, giving five biological donor samples in total. Fresh cecal digesta from donors was suspended in pre-cooled sterile normal saline, filtered through sterile gauze to remove coarse particulate debris, and administered within 1 h after collection to minimize viability loss. No frozen storage was applied to the raw FMT material.

The samples were serially diluted to 10^−4^, 10^−5^ and 10^−6^, and 0.1 mL of each dilution was spread on MRS and LB solid medium plates; MRS was applied for isolating lactic-acid bacteria, while LB medium was used to recover facultative anaerobic bacilli. The plates were incubated in an anaerobic incubator (N_2_:CO_2_:H_2_ = 85:10:5) at 37 °C for 48–72 h. Single colonies with lactobacillus characteristics were picked and purified by streak culture 3–4 times.

The purified strains were identified by Gram staining and 16S rRNA gene sequencing. For 16S rRNA sequencing, bacterial DNA was extracted, and PCR amplification was performed using universal primers (27F: 5′-AGAGTTTGATCCTGGCTCAG-3′; 1492R: 5′-GGTTACCTTGTTACGACTT-3′). The PCR products were subsequently sequenced, and the sequences were aligned with the NCBI GenBank database using the BLAST (v2.14.0) tool. A phylogenetic tree was constructed using MEGA 11 software to confirm the taxonomic status of the strains.

### 2.5. Preparation of Fecal Microbiota Substitute

Strains with good growth performance (OD600 ≥ 1.0 after 24 h of anaerobic culture in MRS and LB liquid medium and a colony diameter ≥ 1.0 mm after 48 h of culture on solid medium) and stable genetic characteristics were selected. The strains were cultured in 100 mL MRS and LB liquid medium at 37 °C for 24 h anaerobically to reach a concentration of 10^8^–10^9^ CFU/mL. The bacterial cells were collected by centrifugation, washed twice with sterile normal saline, and resuspended to a concentration of 10^9^ CFU/mL. Multiple strains were mixed in equal proportions, and 20% glycerol was added as a protective agent. The mixture was aliquoted into sterile centrifuge tubes and stored at −80 °C.

### 2.6. Fecal Microbiota Transplantation Experiments

We used ninety one-day-old XBC chicks (n = 30 per group). The whole animal trial lasted 7 days post-hatch. Oral gavage treatments were performed on day 1, day 2 and day 3 after hatching. Tissue and digesta sampling was performed 48 h after the final gavage (on experimental day 5). The chickens in the FMT group were orally administered 0.2 mL of fecal microbiota solution (viable count: 2.8 × 10^9^ CFU/mL) every day for 3 consecutive days. The FMST group was given 0.2 mL of the fecal microbiota substitute preparation (viable count: 3.2 × 10^9^ CFU/mL), and the CK group was given 0.2 mL of sterile normal saline. All the chickens were raised in a standardized animal room with a controlled temperature (28–32 °C), relative humidity (55–65%), given free access to a basal diet and clean water, 16 h of daily light, regular disinfection and routine immunization.

### 2.7. Cecal Microbial Diversity Detection

Ileal tissue and cecal digesta samples were harvested 48 h after the final gavage treatment to capture acute post-intervention intestinal phenotypes.

We collected cecal contents from each replicate pool for 16S rRNA sequencing (n = 3 per group). Cecal contents (1 g) were collected and stored at −80 °C. Microbial total DNA was extracted, and PCR amplification targeting the V3–V4 region of the bacterial 16S rRNA gene was performed using the primers 338F (5′-ACTCCTACGGGAGGCAGCAG-3′) and 806R (5′-GGACTACHVGGGTWTCTAAT-3′). The PCR products were subsequently sequenced on the Illumina (Illumina, San Diego, CA, USA) MiSeq platform. The α diversity (Shannon index, Chao index) and β diversity (PCA based on the Bray–Curtis distance) were analyzed, and the relative abundance of the dominant microbiota at the genus level was calculated.

### 2.8. Detection of the Morphological Structure of the Ileum

Ileal tissue samples (0.5 cm × 0.5 cm) were fixed in 4% paraformaldehyde for 24 h, dehydrated, transparentized, embedded in paraffin, and sectioned into 3 μm thick slices. The slices were stained with hematoxylin–eosin (HE) and observed under an optical microscope. The villus height, crypt depth and the villus height/crypt depth (V/C) ratio were measured using ImageJ (v1.54f) software.

### 2.9. Measurement of Ileal Inflammatory Cytokine Protein Concentrations (ELISA Assays)

Ileal tissue samples (approximately 50 mg) were homogenized with precooled PBS buffer (1:9, *w/v*) on ice. The homogenate was centrifuged at 3000 r/min at 4 °C for 10 min, and the supernatant was further centrifuged at 12,000 r/min at 4 °C for 15 min. The final supernatant was collected and stored at −20 °C. The levels of IL-6, TNF-α and IL-1β were detected using ELISA kits (Coibo, Shanghai, China) according to the manufacturer’s instructions.

### 2.10. Gene Expression Analysis by Quantitative Real-Time PCR (Inflammatory-Related mRNA Transcripts)

Total RNA was extracted from ileal tissue using TRIzol reagent and reverse transcribed into cDNA. Quantitative PCR (qPCR) was performed using specific primers for IL-6, TNF-α, IL-1βand GAPDH (Table 1). The qPCR system included 5.0 μL of ArtiCanATM SYBR qPCR Mix, 0.2 μL of forward primer, 0.2 μL of reverse primer, 0.2 μL of 50× ROX Reference Dye I, 1.0 μL of template cDNA and 3.4 μL of ddH2O. The reaction conditions were 95 °C for 60 s, followed by 40 cycles of 95 °C for 10 s and 60 °C for 20 s. The relative expression level of genes was calculated using the 2^−ΔΔCT^ method.

### 2.11. Western Blot Analysis of Tight-Junction and Apoptosis-Related Protein Abundance

Protein samples were prepared from ileal tissue, and the protein concentration was determined using a BCA kit (Beyotime, Shanghai, China). The protein samples were separated by SDS–PAGE and transferred to PVDF membranes. The membrane was blocked with 5% skim milk in TBST for 2 h, incubated with primary antibodies (claudin-1, ZO-1, caspase-3, and β-actin; Proteintech, Wuhan, China) at 4 °C overnight, and then incubated with secondary antibodies (Proteintech, Wuhan, China) at 37 °C for 2 h. The protein bands were visualized using an enhanced chemiluminescence (ECL) kit (Beyotime, Shanghai, China).

### 2.12. Statistical Analysis

All the data were analyzed using SPSS 26.0 software and are expressed as the mean ± standard deviation (mean ± SEM). One-way analysis of variance (ANOVA) was used for comparisons between groups, and Duncan’s multiple comparison method was used for post hoc tests. *p* < 0.05 was considered to indicate statistical significance.

## 3. Results

### 3.1. Species Number in Each Intestinal Segment of XBCs

The species numbers of different intestinal segments are shown in Table 2 and are in the order of cecum group (D) > rectum group (E) > jejunum group (B) > ileum group (C) > duodenum group (A). The cecum group had the greatest mean species number (7593.2), with a small standard deviation (187.2), indicating high and stable species richness. The mean number of species in the duodenum group was the lowest (2700.4), with a large standard deviation (1160.3), and significant differences were detected among the samples in the group.

### 3.2. Dominant Species Distribution

**Figure 1:**
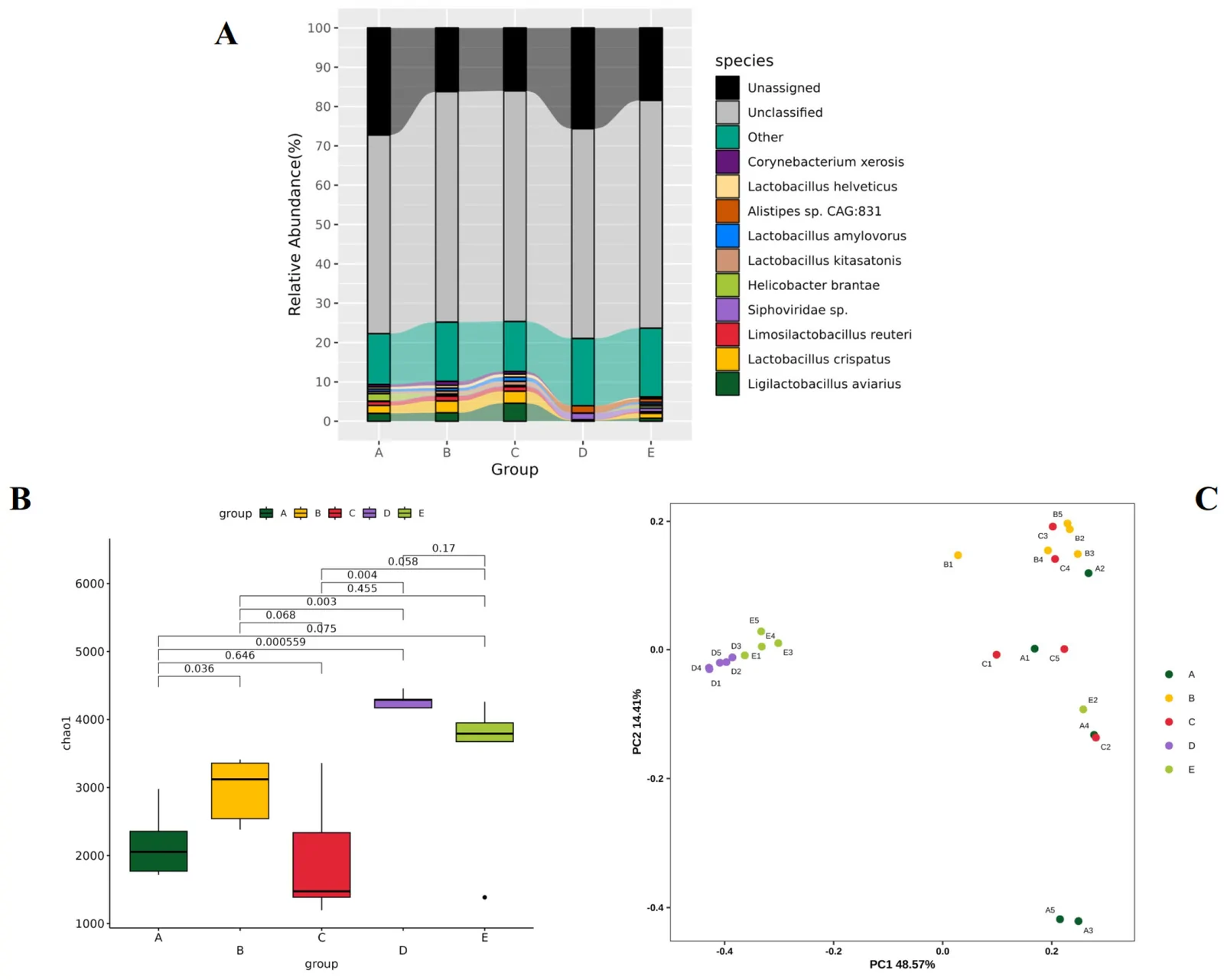
The *α*, *β* diversity and dominant species of various intestinal segments of XBCs. (**A**) The dominant species in the intestine of XBCs; (**B**) the *α* diversity analyses of various intestinal segments of XBCs; (**C**) the *β* diversity analyses of various intestinal segments of XBCs.

The dominant species in the intestine of XBCs are shown in Figure 1A, including *Corynebacterium xerosis*, *Lactobacillus helveticus*, *Alistipes* sp. CAG:831, *Lactobacillus amylovorus*, *Lactobacillus kitasatonis*, *Helicobacter brantae*, *Siphoviridae* sp., *Limosilactobacillus reuteri*, *Lactobacillus crispatus* and *Ligilactobacillus aviarius*. Among them, *Corynebacterium xerosis*, *Alistipes* sp. CAG:831 and *Helicobacter brantae* were the opportunistic strains, while *Lactobacillus helveticus*, *Lactobacillus kitasatonis*, *Limosilactobacillus reuteri*, *Lactobacillus crispatus* and *Ligilactobacillus aviarius* were frequently associated with beneficial gut functions.

### 3.3. α and β Diversity Analysis

The *α* and *β* diversity analyses of various intestinal segments of XBCs are shown in Figure 1B,C. The *α* diversity analysis revealed that the cecum group (D) and rectum group (E) had the highest species richness, whereas the ileum group (C) had the lowest, with significant differences among most groups. *β* diversity PCoA revealed that the samples in groups D and E were closely clustered, indicating a similar microbiota structure. The microbiota structures of groups D and E were significantly different from those of groups A, B and C, indicating obvious grouping characteristics.

### 3.4. Isolation and Identification of Probiotic Strains

Hundreds of visible colonies were picked based on morphological features. In total, eight pure strains were isolated from the cecal contents through anaerobic culture and streak purification. Through 16S rRNA gene sequencing and phylogenetic analysis, three probiotic strains were identified, namely, *Lactobacillus crispatus*, *Weissella paramesenteroides* and *Bacillus amyloliquefaciens* (Supplementary data S1). In addition, two pathogenic strains (*Klebsiella pneumoniae* and *Staphylococcus epidermidis*) were identified, which were not recommended for the preparation of a fecal microbiota solution.

### 3.5. Effects of FMT on the Diversity of XBC Cecal Microbiota

The results of the α diversity analysis are shown in Table 3, indicating that the observed species number and Chao1 index of the FMT group were the highest, at 12,579 and 22,369, respectively, which were higher than those of the FMST group (11,731 and 21,536) and the CK group (12,367 and 21,697, respectively). The Shannon indices of the FMT and CK groups were similar (≈5.97) and significantly greater than that of the FMST group (5.77). The Simpson indices of all three groups were close to 1 (>0.985), and the Pielou_J index of the FMT group was the highest (0.641), indicating the most uniform species distribution.

**Figure 2:**
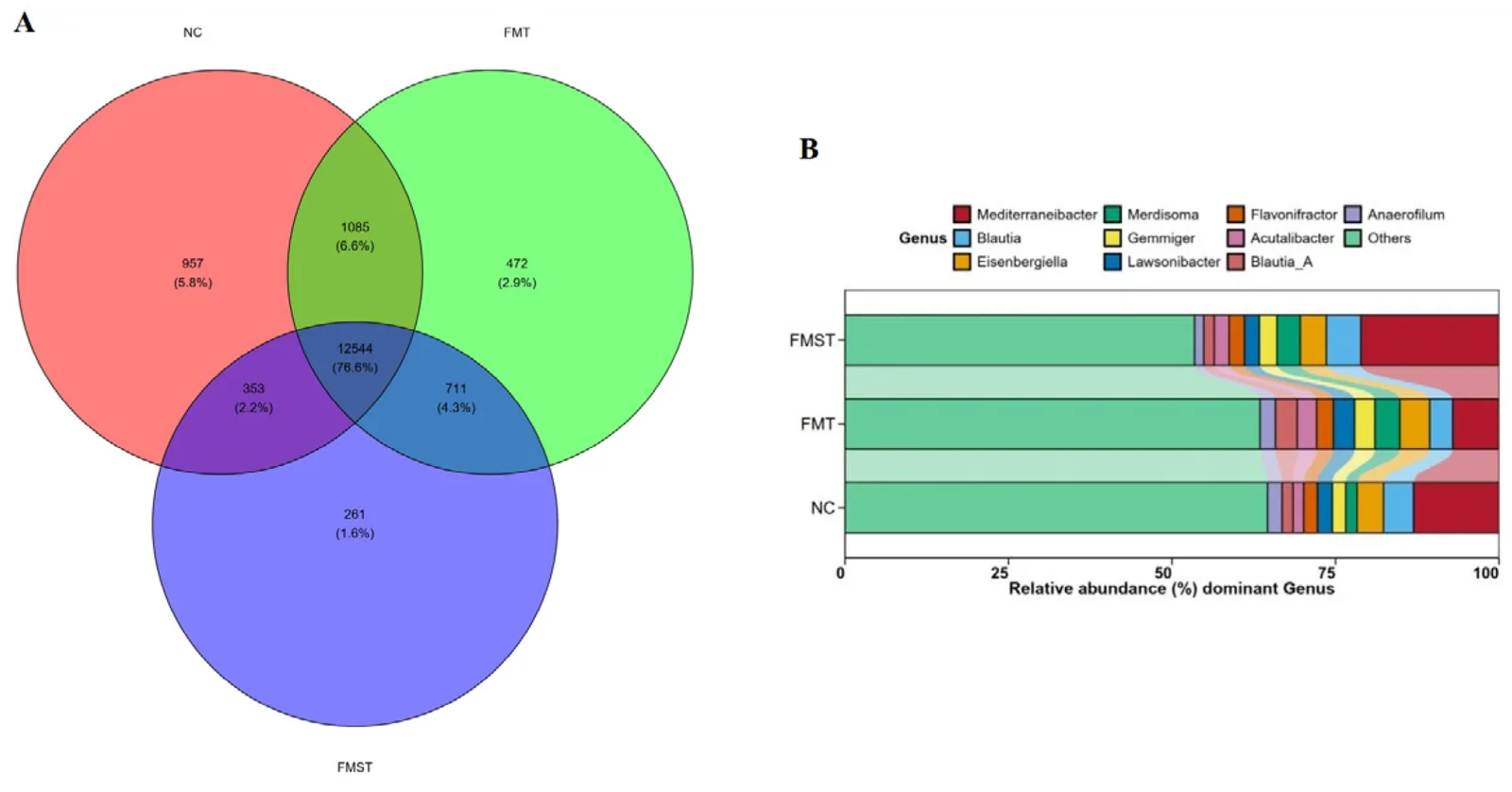
The effect of FMT on the differential distribution of cecal microbiota in XBCs. (**A**) Venn diagram of core species in different groups of XBCs; (**B**) PCoA analysis of beta diversity of cecal microbiota in different groups of XBCs.

Venn diagram analysis (Figure 2A) revealed that the core species of the three groups accounted for more than 76% of the total species. The CK group had the most unique species (957, 5.8%), followed by the FMT group (472, 2.9%) and the FMST group (261, 1.6%). The dominant genera in the cecum included Mediterraneibacter and Blautia (Figure 2B). Compared with the FMT group, the FMST group had a higher abundance of beneficial genera such as Blautia.

### 3.6. Ileal Morphological Structure

**Figure 3:**
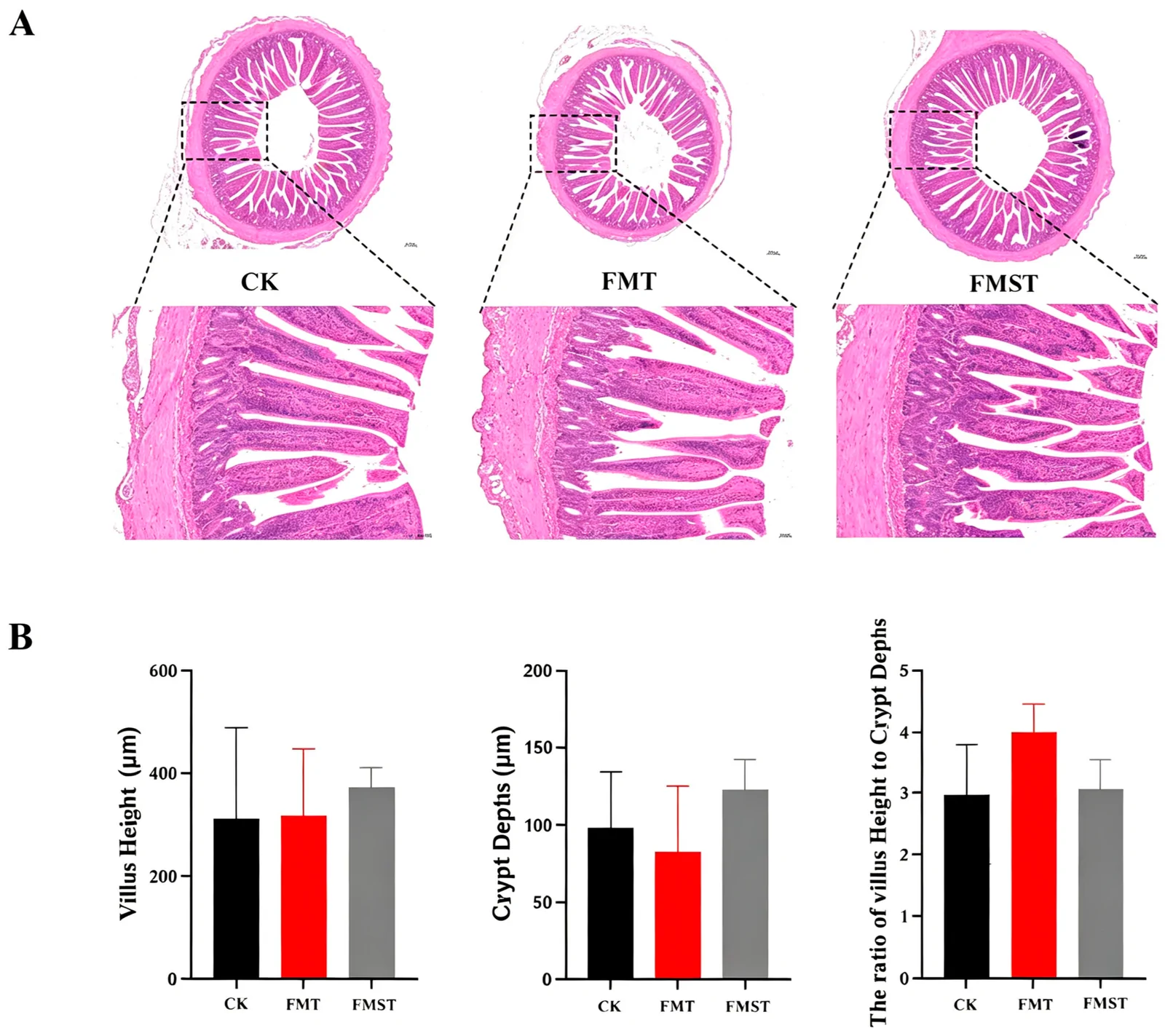
The effect of FMT on the morphological structure of ileum in XBCs. (**A**) HE staining images of ileal tissues from different groups of XBCs; (**B**) morphology of ileal tissues from different groups of XBCs.

HE staining results (Figure 3A) revealed that the ileal villi of most samples in the FMT group and FMST group were regularly arranged, with intact epithelial cells and clear crypt structures with no obvious inflammatory cell infiltration. Analysis of the V/C ratio revealed that the FMT group had the highest V/C ratio, indicating that the intestinal absorption ability and cell proliferation state were most strongly balanced, but the difference was not significant.

### 3.7. Ileal Inflammatory Factor Levels

**Figure 4:**
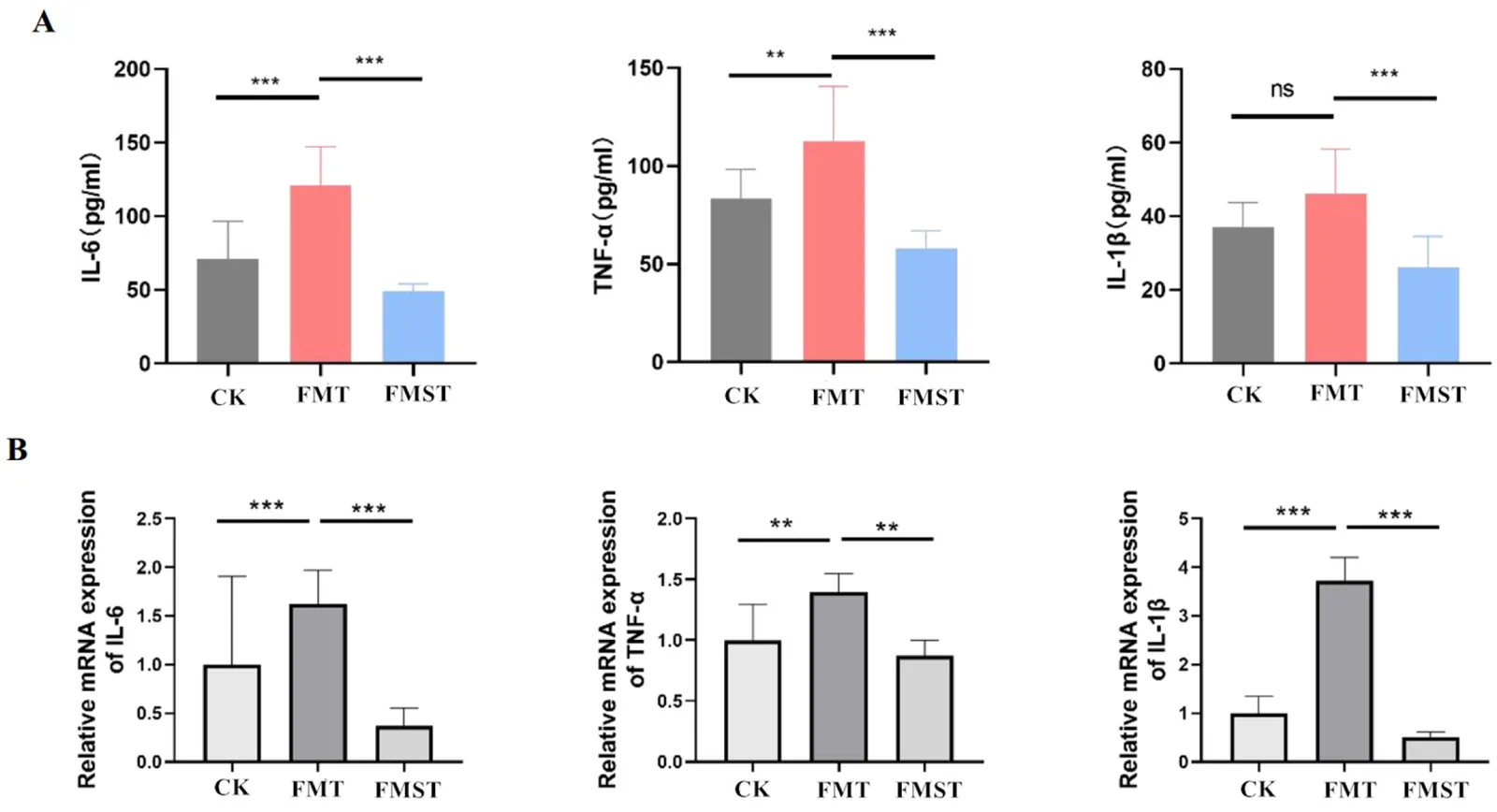
The effect of FMT on the levels of inflammatory factors in the ileum of XBCs. (**A**) Levels of pro-inflammatory cytokines in the ileum from different groups of XBCs; (**B**) mRNA expression levels of pro-inflammatory cytokines in the ileum from different groups of XBCs. Statistical significance is indicated as follows: ns *p* > 0.05, ** *p* < 0.01, *** *p* < 0.001.

The ELISA results (Figure 4A) revealed that the levels of pro-inflammatory cytokines (IL-6 and TNF-α) in the FMT group were significantly greater than those in the CK and FMST groups, indicating an obvious subclinical inflammatory response in the intestine. The levels of pro-inflammatory cytokines in the FMST group were lower than those in the FMT and CK groups.

The qPCR results (Figure 4B) revealed that the mRNA expression levels of TNF-α, IL-1β and IL-6 in the FMT group were significantly greater than those in the CK and FMST groups.

### 3.8. Expression of Tight Junction Proteins

**Figure 5:**
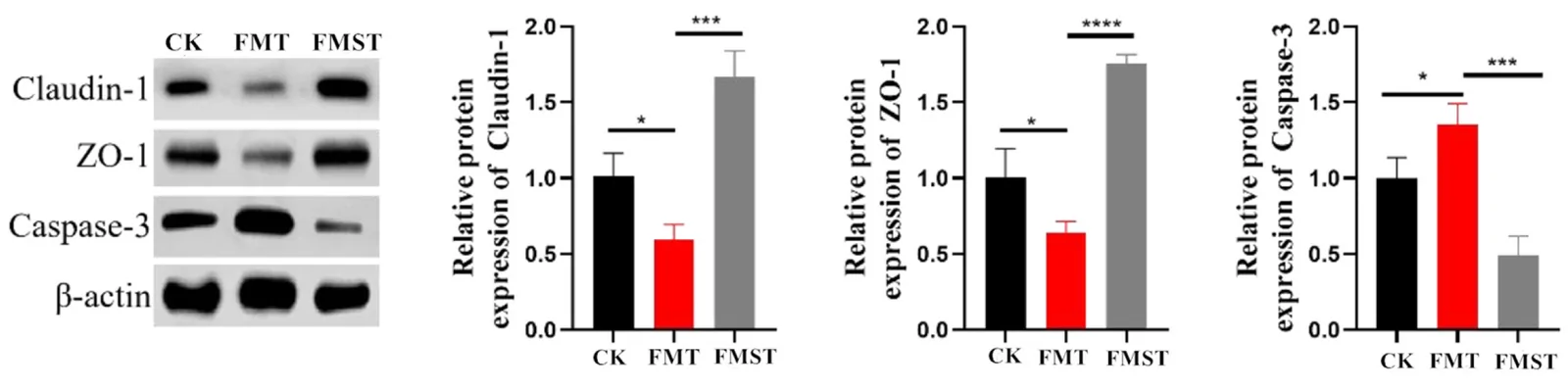
The effect of FMT on the expression of tight junction proteins in the ileum of XBCs. Statistical significance is indicated as follows: * *p* < 0.05, *** *p* < 0.001, **** *p* < 0.0001.

The Western blot results (Figure 5) revealed that the protein expression levels of claudin-1 and ZO-1 in the FMT group were significantly lower than those in the CK and FMST groups, while the protein expression level of caspase-3 was significantly greater.

## 4. Discussion

As the main site of microbial fermentation in poultry, the cecum has a relatively slow digesta transit rate and an anaerobic environment, providing a stable niche for the colonization and reproduction of a variety of microorganisms, especially those involved in the metabolism of indigestible carbohydrates [10]. In contrast, the small intestine is the main site of nutrient absorption in chickens, with a fast digesta transit rate and a relatively aerobic environment, which limits the survival of anaerobic microorganisms, resulting in lower species richness [11]. The results of this study revealed that the species richness of the intestinal microbiota of XBCs exhibited a distinct segmental distribution pattern, with the cecum having the highest species number and the duodenum having the lowest, and the microbiota structure of the cecum and rectum was highly similar but significantly different from that of the small intestine (duodenum, jejunum, and ileum), adult cecal microbial profiles were used only as a resource for isolating candidate strains. Five biological replicates could not fully eliminate individual microbiota variation in indigenous chickens. These findings are consistent with the basic physiological characteristics of avian intestinal microbiota and are supported by many poultry microbiota studies [11,12,13]. Compared with standard chickens, black-bone chickens are widely valued for their medicinal value, and black-bone chickens can be used for disease prevention and control [14]. Microbiomics analysis of the gut microbiota of Lueyang black bone chickens revealed significant differences in microbial communities between the duodenum and cecum at the genus level. LEfSe analysis revealed seven characteristic genera in the duodenum (e.g., *Bacteroides* and *Alistipes*) and five in the cecum (e.g., *Lactobacillus* and *Ureaplasma*) [15].

The disease resistance of chickens is influenced by many environmental factors, and differences in disease resistance exist among different chicken breeds and strains [16]. Native chicken breeds are more resistant to diseases, especially bacterial diseases, than exotic chicken breeds, which face extensive selection pressure in terms of production traits [17]. The survival rate and average time of death of Nicobari chickens, a local chicken breed, were significantly greater than those of Vanaraja chickens, a hybrid improved breed, during experimental Pasteurella infection [18]. In recent years, the exploration of native chicken immunity and its underlying mechanisms has been a global focus of attention. The gut microbiota is crucial for the health, disease, growth, and egg production of chickens [19,20,21]. The meat and egg production quality of native chickens such as Jianghan chickens is better than that of centralized chickens, and the diversity of the intestinal microbiota is greater. Researchers have screened more than 829 isolates from the microbial community of Jianghan chickens, and three of these strains have shown strong probiotic potential. Therefore, potential intestinal microbiota resources may benefit host health. Significant differences were observed in the cecal microbes: Bacteroides was significantly enriched in the White Leghorn (a well-known commercial layer line chicken with a high egg production rate), whereas Veillonellaceae and Parabacteroides were significantly enriched in Silky Fowl (a Chinese native chicken variety that has a low egg production rate but good immune performance) [22]. Studies have shown that compared with Arbor Acre broilers, Chinese native Wuliang Mountain black-bone chickens have stronger mucosal immunity, including higher levels of IgA, a more diverse IgA antibody library, and higher bacterial affinity. Bacteroides has been identified as a key microorganism associated with the intestinal IgA response [23]. This study revealed that the dominant microbiota in the intestine of XBCs included both strains frequently associated with beneficial gut functions in poultry (e.g., *Lactobacillus helveticus*, *Lactobacillus crispatus*, and *Ligilactobacillus aviarius*) and opportunistic strains (e.g., *Corynebacterium xerosis* and *Helicobacter brantae*). The co-existence of probiotics and pathogenic bacteria results in a dynamic balance in the intestinal tract, which is a key feature of the healthy intestinal microbiota of poultry [24]. The identified microbiota may provide candidate microbial resources for future studies of XBC health traits, which can inhibit the overproliferation of pathogenic bacteria through mechanisms such as nutrient competition, colonization resistance, and the production of antimicrobial metabolites (e.g., lactic acid and hydrogen peroxide) [25]. Therefore, through LB and MRS liquid medium culture and 16S rRNA gene sequencing, three probiotic strains were successfully screened from the cecal contents of XBCs: *Lactobacillus crispatus*, *Weissella paramesenteroides*, and *Bacillus amyloliquefaciens*. These species have been reported to possess probiotic potential.

*Lactobacillus crispatus* is a typical lactic acid-producing bacterium that is widely distributed in the intestinal tract of healthy poultry. One study showed that *Lactobacillus crispatus* supplementation can significantly reduce the levels of pro-inflammatory cytokines (IL-1β, IL-6, TNF-α) in the oviduct and intestinal tract of laying hens, mitigate inflammatory damage to the intestinal epithelial barrier, and increase the abundance of beneficial bacteria in the cecum [26]. In addition, *Lactobacillus crispatus* can competitively adhere to the intestinal mucosal surface, reduce the adhesion rate of pathogens, and enhance mucosal immunity, which is important for its ability to improve intestinal health [27]. *Weissella paramesenteroides* is a lactobacillus with unique stress resistance and metabolic characteristics. Kinjal Pabari’s study confirmed that this strain can survive under harsh conditions, such as low pH (2.5) and high salt concentration (7.0–13.0%), and has a strong prebiotic utilization ability, which can decompose galactooligosaccharides and fructooligosaccharides to produce large amounts of short-chain fatty acids [28]. Therefore, its high salt tolerance and heat resistance make it more suitable for the complex intestinal environment of chickens and the industrial production process of microbial preparations. During a screening program for bacteriocin producers, a new lactic acid bacterium isolated from the chicken gizzard was reported to produce a bacteriocin that inhibited Gram-positive and Gram-negative food-borne pathogens, especially Salmonella typhimurium, according to 16S rRNA gene nucleotide sequencing and phylogenetic analysis, and the newly isolated strain was *Weissella paramesenteroides* [29]. In addition to improving the histopathological appearance of the colon, *Weissella paramesenteroides* ingestion improved the health of mice with colitis and reduced intestinal permeability [30]. In addition to these two strains of lactic acid bacteria, the *Bacillus amyloliquefaciens* strains isolated in this study are widely used as alternatives to antibiotics in poultry production. *Bacillus amyloliquefaciens* is a Gram-positive facultative anaerobic bacterium that is capable of producing a range of metabolites that inhibit fungal and bacterial activity during its growth process [31]. Research has shown that *Bacillus amyloliquefaciens* can improve the production performance of broiler chickens and significantly increase their potential for the biosynthesis of amino acids and vitamins in the cecal microbiome [32]. In vitro experiments revealed that *Bacillus amyloliquefaciens* isolated from healthy chicken intestines inhibited the growth of *Listeria monocytogenes* and Methicillin-resistant *Staphylococcus aureus* [33]. Researchers have also reported that newly isolated *Bacillus amyloliquefaciens* significantly increased breast muscle production in broiler chickens, improved jejunal morphology, as evidenced by increased villus height and the villus height-to-crypt depth ratio, and positively modulated the cecal microbiota composition [34]. These findings indicate that *Bacillus amyloliquefaciens* has strong probiotic properties and can promote the growth performance and intestinal health of broiler chickens, demonstrating its feasibility as a substitute for antibiotic growth promoters. Therefore, in this study, these three probiotic strains were selected to prepare replacements for the fecal microbiota, and comparative experiments with FMT were conducted. While these three species have been widely reported to carry probiotic-associated properties in previous poultry studies, dedicated strain-level functional and safety assays for our isolates remain to be completed in future work.

This study revealed that traditional FMT can improve the α diversity of the cecal microbiota of Xichuan black-bone chickens (the observed species number and Chao1 index were the highest), but it also induces a significant subclinical inflammatory response in the intestinal tract, characterized by a significant increase in the levels of pro-inflammatory cytokines (IL-6, TNF-α, and IL-1β) and a decrease in the expression of intestinal tight junction proteins (Claudin-1 and ZO-1), while the expression of caspase-3, an apoptosis-related protein, is significantly upregulated. This phenomenon is closely related to the characteristics of unoptimized FMT. Although conventional FMT achieved higher cecal microbial α-diversity indices in our trial, this increased diversity was accompanied by measurable subclinical intestinal inflammatory phenotypes. FMST improved intestinal barrier-related molecular markers without restoring FMT-like high microbial community diversity. Therefore, FMST does not microbiologically recapitulate the FMT community; its benefits appear to be driven by host intestinal immune-barrier modulation rather than reconstruction of high-diversity cecal microbiota. During the FMT process, it might be somewhat difficult to detect pathogens and transfer drug-resistant bacteria [35]. Unmodified FMT fecal microbiota solutions may contain small amounts of pathogenic bacteria, harmful metabolites, or undigested feed residues, which can activate the host’s innate immune response after transplantation, leading to the upregulation of pro-inflammatory cytokine expression and the occurrence of mild intestinal inflammation [36]. Although this subclinical inflammation does not cause obvious pathological damage to intestinal tissue (no obvious villus atrophy or inflammatory cell infiltration was observed by HE staining), it can still damage intestinal barrier function. Research on AA broiler chickens has shown that FMT rapidly regulates the intestinal flora but has little effect on intestinal function and a greater potential risk of damage to the jejunum [37]. In contrast, FMST prepared by screening probiotic strains effectively solved the above problems associated with FMT. The results showed that FMST could reduce the level of intestinal subclinical inflammation, significantly downregulate the expression of pro-inflammatory cytokines, and upregulate the protein expression of claudin-1 and ZO-1 while reducing the expression of caspase-3. Conventional FMT decreased the relative abundance of Mediterraneibacter and Blautia, whereas FMST significantly enriched them; this correlation may partially explain the phenotypic differences. We hypothesize that the key factor underlying the significant differences in intestinal inflammatory cytokines, tight junction proteins, and apoptotic proteins between the FMST and FMT groups was the divergent regulation of Mediterraneibacter and Blautia abundance.

Blautia, a core commensal genus within the phylum Firmicutes, has been repeatedly confirmed to be associated with gut health [38,39,40]. As a key beneficial bacterium, Blautia is strongly positively correlated with intestinal barrier function, and its enrichment by Portulaca oleracea L. extract (POLE) helps enhance gut barrier integrity, reduce pro-inflammatory cytokine levels, and mitigate apoptosis, all of which play vital roles in maintaining intestinal health in chickens [40]. Blautia also contributes to maintaining a balanced gut microbiota and plays an important role in mitigating heat-stress-related intestinal disorders in broilers [41] and helps to mitigate LPS-induced intestinal injury and inflammation in broilers [42]. Blautia was identified as a key bacterium positively associated with intestinal health in chickens, and higher Blautia abundance correlated with lower intestinal inflammation and improved feed efficiency [43]. Mechanistically, Blautia produces substantial amounts of short-chain fatty acids (SCFAs), particularly acetate and propionate, which inhibit the NF-κB signaling pathway, reduce the release of pro-inflammatory mediators, and reinforce tight junction assembly [44]. Furthermore, SCFAs generated by Blautia suppress intestinal epithelial apoptosis by downregulating the expression of pro-apoptotic proteins such as caspase-3, thus preserving mucosal structure and function [45]. Mediterraneibacter, another Firmicutes member, is negatively associated with intestinal inflammation and is regarded as a biomarker of healthy cecal microbiota [46]. In a necrotic enteritis model, the severity of intestinal lesions was accompanied by a progressive decrease in the abundance of Mediterraneibacter, indicating its role in resisting pathogenic invasion and maintaining microbial homeostasis [47]. Mediterraneibacter participates in carbohydrate fermentation and SCFA production, which synergize with Blautia to create an anti-inflammatory microenvironment, stabilize the gut barrier, and reduce epithelial apoptosis [48]. Collectively, the co-enrichment of Mediterraneibacter and Blautia represents a microbial signature indicative of enhanced intestinal health. In the current study, FMST significantly increased the abundance of Mediterraneibacter and Blautia, which consequently downregulated the mRNA and protein levels of IL-6, TNF-α, and IL-1β; upregulated claudin-1 and ZO-1 expression; and reduced caspase-3 activation. These findings are consistent with reports that enrichment of SCFA-producing commensals strengthens barrier function and mitigates inflammation. SCFA measurement is required in future work to test this hypothetical pathway. In contrast, conventional FMT reduced the levels of these two beneficial genera, leading to aggravated subclinical inflammation, impaired tight junctions, and increased epithelial apoptosis. This unfavorable outcome likely arises from the complex and unstandardized composition of the raw fecal microbiota, which may contain pathobionts or pro-inflammatory components that displace beneficial commensals and disrupt the microbial balance [49], the underlying causal mechanism requires further direct validation.

## 5. Conclusions

Overall, the cecum of XBCs has the most abundant microbial resources, and the FMST shows great potential to serve as a safer standardized alternative to traditional FMT under current experimental conditions. We hypothesize that the divergent regulation of Mediterraneibacter and Blautia may be associated with the observed phenotypic differences underlying the phenotypic differences between FMST and FMT. FMST-mediated enrichment of these two genera enhances SCFA production, suppresses inflammatory signaling, strengthens tight junction assembly, and reduces intestinal epithelial apoptosis, further targeted metabolomic and in vitro cell trials are required to validate this causal mechanism. This correlation suggests potential regulatory effects, targeted modulation of beneficial commensals represents a reliable and safer strategy to improve intestinal health in poultry, supporting the application of standardized FMST as a superior alternative to conventional FMT. The current study ignored sex-based differences in intestinal microbiota and intestinal immune responses. We proposed that separate sex grouping and stratified microbiota/immune testing should be performed in follow-up research to dissect sex-specific regulatory patterns of FMT/FMST.

## Figures and Tables

**Table 1:** Sequences of the primers used for quantitative real-time PCR.

Gene Name	Sequence (5′~3′)	Product Size, bp	Accession Number
IL-6	F-ATAAATCCCGATGAAGTGGTC	143	NM_204628.2
	R-CACGGTCTTCTCCATAAACG		
IL-1β	F-TGCTGGTTTCCATCTCGTAT	127	NM_204524.2
	R-ACGGGACGGTAATGAAACA		
TNF-α	F-CCCTACCCTGTCCCACAA	147	NM_204267.2
	R-GGCGGTCATAGAACAGCAC		
GAPDH	F-GGACCAGGTTGTCTCCTGTG	151	XM_049824763.1
	R-TCCTTGGATGCCATGTGGAC		

**Table 2:** The number of species groups of contents in each intestinal segment of XBCs.

Category	A	B	C	D	E
Kingdom	7.0 ± 1.6	7.6 ± 0.9	8.2 ± 1.3	8.4 ± 0.5	8.2 ± 1.1
Phylum	53.0 ± 13.2	77.4 ± 11.8	67.8 ± 12.5	146.4 ± 2.3	116.6 ± 35.5
Class	64.4 ± 13.5	83.8 ± 10.6	83.2 ± 8.7	149.4 ± 2.7	121.4 ± 32.5
Order	133.8 ± 25.9	179.0 ± 16.9	172.0 ± 20.3	296.8 ± 3.3	241.6 ± 62.5
Family	249.0 ± 47.5	352.0 ± 29.7	329.2 ± 43.3	596.6 ± 7.6	478.8 ± 133.6
Genus	879.4 ± 224.1	1348.8 ± 72.6	1157.2 ± 173.5	2040.0 ± 54.4	1702.6 ± 477.3
Species	2700.4 ± 1160.3	5525.8 ± 336.0	4392.0 ± 961.5	7593.2 ± 187.2	6647.0 ± 1940.3

Data are expressed as mean ± standard error of the mean (SEM) with n = 5 per group.

**Table 3:** Alpha diversity of XBC cecal microbiota.

Items	CK	FMT	FMST	ANOVA *p*
Observed species	12,367 ± 1015	12,579 ± 645	11,731 ± 292	0.416
Chao1	21,697 ± 2386	22,369 ± 3718	21,536 ± 305	0.916
ACE	20,595 ± 2302	21,052 ± 3235	20,611 ± 238	0.965
Shannon	5.96 ± 0.15	5.97 ± 0.10	5.77 ± 0.24	0.375
Simpson	0.989 ± 0.003	0.989 ± 0.003	0.985 ± 0.007	0.508
Pielou_J	0.633 ± 0.011	0.641 ± 0.014	0.617 ± 0.021	0.458

Data are expressed as mean ± standard error of the mean (SEM) with n = 3 per group.

## Data Availability

The original contributions presented in this study are included in the article/Supplementary Materials. Further inquiries can be directed to the corresponding author.

## Supplementary Materials

The following supporting information can be downloaded at: https://www.mdpi.com/article/10.3390/microorganisms14091966/s1, Supplementary data S1: Blast (NCBI Comparison and Identification Table Results). Supplementary data S2: metagenomic sequencing details. Supplementary data S3: HE and WB raw data.

## References

[1] Li D., Sun G., Zhang M., Cao Y., Zhang C., Fu Y., Li F., Li G., Jiang R., Han R. (2020). Breeding History and Candidate Genes Responsible for Black Skin of Xichuan Black-Bone Chicken. BMC Genom..

[2] Li D., Wang X., Fu Y., Zhang C., Cao Y., Wang J., Zhang Y., Li Y., Chen Y., Li Z. (2019). Transcriptome Analysis of the Breast Muscle of Xichuan Black-Bone Chickens under Tyrosine Supplementation Revealed the Mechanism of Tyrosine-Induced Melanin Deposition. Front. Genet..

[3] Ren J., He Q., Shang H., Lu T., Xiong X. (2026). Host Genetic and Environmental Determinants of Chicken Gut Microbiota: A Review. Poult. Sci..

[4] Chen S., Liu T., Chen J., Shen H., Wang J. (2025). Fecal Virome Transplantation Confirms Non-Bacterial Components (Virome and Metabolites) Participate in Fecal Microbiota Transplantation-Mediated Growth Performance Enhancement and Intestinal Development in Broilers with Spatial Heterogeneity. Microorganisms.

[5] Gao C., Chen Y., Zhang Z., Xu D., Liu X., Wang D., Shi L., Wang X., Chen H., Hao E. (2025). Laying Rate Was Correlated with Microbial Fecal Microbiota Transplantation Improves the Laying Performance by Changing the Gut Microbiota Composition in Late Laying Period. Poult. Sci..

[6] Zhang Q., Zhu Q., Xiao Y., Liao S., Liu S., Shi S. (2026). Microbiota-Derived Propionate Suppresses Salmonella Virulence Gene Expression Via Luxs Quorum Sensing. Microbiome.

[7] Ramesh A., Subbarayan R., Shrestha R., Adtani P.N. (2026). Exploring Fecal Microbiota Transplantation: Potential Benefits, Associated Risks, and Challenges in Cancer Treatment. Cancer Rep..

[8] Thanush D., Venkatesh M.P. (2023). Fecal Microbiota Transplantation: History, Procedure and Regulatory Considerations. Presse Med..

[9] Liu C.S., Merrick B., Taboun Z.S., Mullish B.H., Goldenberg S.D., Terveer E.M., Porcari S., Bradbury R.S., Ianiro G., Ng S.C. (2025). Towards Optimising and Standardising Donor Screening for Faecal Microbiota Transplantion. Gut.

[10] Deryabin D., Lazebnik C., Vlasenko L., Karimov I., Kosyan D., Zatevalov A., Duskaev G. (2024). Broiler Chicken Cecal Microbiome and Poultry Farming Productivity: A Meta-Analysis. Microorganisms.

[11] Ye D., Qiu J., Fan Z., Zhu L., Lv C., Guo P. (2025). Region-Specific Gut Microbiome Variation between Changle Geese and Yellow-Feathered Broilers: Correlations with Growth and Intestinal Development. Microorganisms.

[12] Stamilla A., Ruiz-Ruiz S., Artacho A., Pons J., Messina A., Randazzo C.L., Caggia C., Lanza M., Moya A. (2021). Analysis of the Microbial Intestinal Tract in Broiler Chickens During the Rearing Period. Biology.

[13] Yan W., Sun C., Zheng J., Wen C., Ji C., Zhang D., Chen Y., Hou Z., Yang N. (2019). Efficacy of Fecal Sampling as a Gut Proxy in the Study of Chicken Gut Microbiota. Front. Microbiol..

[14] Khumpeerawat P., Duangjinda M., Phasuk Y. (2021). Factors Affecting Gene Expression Associated with the Skin Color of Black-Bone Chicken in Thailand. Poult. Sci..

[15] Zhao M., Zeng S., Shao L., Wang L., Zhang T., Lu H., Zeng W. (2025). Antibiotic Residues in Muscle Tissues of Lueyang Black-Bone Chickens under Free-Range Mountainous Conditions and Their Association with Gut Microbiota. Microorganisms.

[16] Zekarias B., Huurne A.A.T., Landman W.J., Rebel J.M., Pol J.M., Gruys E. (2002). Immunological Basis of Differences in Disease Resistance in the Chicken. Vet. Res..

[17] Jeong H.S., Kim D.W., Chun S.Y., Sung S., Kim H.J., Cho S., Kim H., Oh S.J. (2014). Native Pig and Chicken Breed Database: Npcdb. Asian-Australas. J. Anim. Sci..

[18] Kannaki T.R., Priyanka E., Haunshi S. (2021). Research Note: Disease Tolerance/Resistance and Host Immune Response to Experimental Infection with Pasteurella Multocida A:1 Isolate in Indian Native Nicobari Chicken Breed. Poult. Sci..

[19] Wickramasuriya S.S., Park I., Lee K., Lee Y., Kim W.H., Nam H., Lillehoj H.S. (2022). Role of Physiology, Immunity, Microbiota, and Infectious Diseases in the Gut Health of Poultry. Vaccines.

[20] Pangga G.M., Star-Shirko B., Psifidi A., Xia D., Corcionivoschi N., Kelly C., Hughes C., Lavery U., Richmond A., Ijaz U.Z. (2025). Impact of Commercial Gut Health Interventions on Caecal Metagenome and Broiler Performance. Microbiome.

[21] Gong H., Liang F., Cai C., Ding X., Bai S., Zhang K., Zeng Q., Liu Y., Xuan Y., Xu S. (2025). Dietary Saccharomyces Cerevisiae Fermentation Product Improved Egg Quality by Modulating Intestinal Health, Ovarian Function, and Cecal Microbiota in Post-Peak Laying Hens. Poult. Sci..

[22] Yang X., Tai Y., Ma Y., Xu Z., Hao J., Han D., Li J., Deng X. (2022). Cecum Microbiome and Metabolism Characteristics of Silky Fowl and White Leghorn Chicken in Late Laying Stages. Front. Microbiol..

[23] Wang X., Hu Y., Zhu X., Cai L., Farooq M.Z., Yan X. (2023). Bacteroides-Derived Isovaleric Acid Enhances Mucosal Immunity by Facilitating Intestinal Iga Response in Broilers. J. Anim. Sci. Biotechnol..

[24] Reuben R.C., Roy P.C., Sarkar S.L., Alam R.U., Jahid I.K. (2019). Isolation, Characterization, and Assessment of Lactic Acid Bacteria toward Their Selection as Poultry Probiotics. BMC Microbiol..

[25] Khurajog B., Disastra Y., Lawwyne L.D., Sirichokchatchawan W., Niyomtham W., Yindee J., Hampson D.J., Prapasarakul N. (2023). Selection and Evaluation of Lactic Acid Bacteria from Chicken Feces in Thailand as Potential Probiotics. PeerJ.

[26] Li S., Wang Q., Mi J., Chen H., Yuan T., Wang Y., Zhao L., Ma Q., Huang S. (2024). Lactobacillus Crispatus-Mediated Gut-Reproductive Tract Axis-Alleviated Microbial Dysbiosis and Oviductal Inflammation in a Laying Hen Model. Microorganisms.

[27] Edelman S., Leskelä S., Ron E., Apajalahti J., Korhonen T.K. (2003). In Vitro Adhesion of an Avian Pathogenic Escherichia Coli O78 Strain to Surfaces of the Chicken Intestinal Tract and to Ileal Mucus. Vet. Microbiol..

[28] Pabari K., Pithva S., Kothari C., Purama R.K., Kondepudi K.K., Vyas B.R.M., Kothari R., Ambalam P. (2020). Evaluation of Probiotic Properties and Prebiotic Utilization Potential of Weissella Paramesenteroides Isolated from Fruits. Probiotics Antimicrob. Proteins.

[29] Chakchouk-Mtibaa A., Elleuch L., Smaoui S., Najah S., Sellem I., Mejdoub H., Abdelkafi S., Mellouli L. (2014). Characterization of the Bacteriocin Bacj1 and Its Effectiveness for the Inactivation of Salmonella Typhimurium During Turkey Escalope Storage. Food Chem..

[30] Sandes S., Figueiredo N., Pedroso S., Sant’Anna F., Acurcio L., Abatemarco M., Barros P., Oliveira F., Cardoso V., Generoso S. (2020). Weissella Paramesenteroides Wpk4 Plays an Immunobiotic Role in Gut-Brain Axis, Reducing Gut Permeability, Anxiety-Like and Depressive-Like Behaviors in Murine Models of Colitis and Chronic Stress. Food Res. Int..

[31] Zalila-Kolsi I., Ben-Mahmoud A., Al-Barazie R. (2023). Bacillus Amyloliquefaciens: Harnessing Its Potential for Industrial, Medical, and Agricultural Applications-a Comprehensive Review. Microorganisms.

[32] Bajagai Y.S., Yeoh Y.K., Li X., Zhang D., Dennis P.G., Ouwerkerk D., Dart P.J., Klieve A.V., Bryden W.L. (2023). Enhanced Meat Chicken Productivity in Response to the Probiotic Bacillus Amyloliquefaciens H57 Is Associated with the Enrichment of Microbial Amino Acid and Vitamin Biosynthesis Pathways. J. Appl. Microbiol..

[33] Chen J.N., Wei C.W., Liu H.C., Chen S.Y., Chen C., Juang Y.M., Lai C.C., Yiang G.T. (2016). Extracts Containing Clps of Bacillus amyloliquefaciens Jn68 Isolated from Chicken Intestines Exert Antimicrobial Effects, Particularly on Methicillin-Resistant Staphylococcus aureus and Listeria monocytogenes. Mol. Med. Rep..

[34] Tang Y., Yu X., Guo Y., Yue R., Yuan J. (2025). Antimicrobial and Nutritional Potentials of Bacillus Strains. Int. J. Mol. Sci..

[35] Zellmer C., Sater M.R.A., Huntley M.H., Osman M., Olesen S.W., Ramakrishna B. (2021). Shiga Toxin-Producing Escherichia Coli Transmission Via Fecal Microbiota Transplant. Clin. Infect. Dis..

[36] Wu D., Liang S., Du X., Xiao J., Feng H., Ren Z., Yang X., Yang X. (2024). Effects of Fecal Microbiota Transplantation and Fecal Virome Transplantation on Lps-Induced Intestinal Injury in Broilers. Poult. Sci..

[37] Feng H., Xiong J., Liang S., Wang Y., Zhu Y., Hou Q., Yang X., Yang X. (2024). Fecal Virus Transplantation Has More Moderate Effect Than Fecal Microbiota Transplantation on Changing Gut Microbial Structure in Broiler Chickens. Poult. Sci..

[38] Lu Y., Cui A., Zhang X. (2024). Commensal Microbiota-Derived Metabolite Agmatine Triggers Inflammation to Promote Colorectal Tumorigenesis. Gut Microbes.

[39] Wu Y., Ran L., Yang Y., Gao X., Peng M., Liu S., Sun L., Wan J., Wang Y., Yang K. (2023). Deferasirox Alleviates Dss-Induced Ulcerative Colitis in Mice by Inhibiting Ferroptosis and Improving Intestinal Microbiota. Life Sci..

[40] Lun J., Liu M., Zhang W., Huang G., Ma M., Jin W., Zhu Y., Qu Q., Lv W., Guo S. (2025). Influence of Purslane Extract on Immuno-Antioxidant Status, Intestinal Barrier, and Microbiota of Chicks after Experimental Infection with Escherichia Coli O78. Poult. Sci..

[41] Elokil A., Li S., Chen W., Farid O., Abouelezz K., Zohair K., Nassar F., El-Komy E., Farag S., Elattrouny M. (2024). Ethoxyquin Attenuates Enteric Oxidative Stress and Inflammation by Promoting Cytokine Expressions and Symbiotic Microbiota in Heat-Stressed Broilers. Poult. Sci..

[42] Wang X., Zhang T., Li W., Zhang M., Zhao L., Wang N., Zhang X., Zhang B. (2024). Dietary Supplementation with Macleaya Cordata Extract Alleviates Intestinal Injury in Broiler Chickens Challenged with Lipopolysaccharide by Regulating Gut Microbiota and Plasma Metabolites. Front. Immunol..

[43] Chen L., Peng W., Wang T., Ma S., Wang X., Dai B., Zhang R., Yang C., Wu Y. (2025). Effects of Dietary Lysozyme Supplementation on Growth Performance, Intestinal Morphology, Immune Function, Antioxidant Capacity, and Gut Microbiota in Broilers. Poult. Sci..

[44] Ordoñez-Rodriguez A., Roman P., Rueda-Ruzafa L., Campos-Rios A., Cardona D. (2023). Changes in Gut Microbiota and Multiple Sclerosis: A Systematic Review. Int. J. Environ. Res. Public Health.

[45] Mao B., Guo W., Liu X., Cui S., Zhang Q., Zhao J., Tang X., Zhang H. (2023). Potential Probiotic Properties of Blautia Producta against Lipopolysaccharide-Induced Acute Liver Injury. Probiotics Antimicrob. Proteins.

[46] Jones J., García-Martínez K.Y., Lee Y.Y., Rhee M., Nath R.R., Takahashi S., Grodner B., De Vlaminck I., Leifer C.A., Brito I.L. (2025). Gut Commensal Microbiota Drive Tailored Macrophage Responses. Cell Rep..

[47] Yang Q., Liu J., Wang X., Robinson K., Whitmore M.A., Stewart S.N., Zhao J., Zhang G. (2021). Identification of an Intestinal Microbiota Signature Associated with the Severity of Necrotic Enteritis. Front. Microbiol..

[48] Borgonovi T.F., Salgaço M.K., Oliveira G.L.V., Carvalho L.A.L., Pinheiro D.G., Todorov S.D., Sivieri K., Casarotti S.N., Penna A.L.B. (2022). Functional Fermented Milk with Fruit Pulp Modulates the in Vitro Intestinal Microbiota. Foods.

[49] Merrick B., Allen L., Masirah M.Z.N., Forbes B., Shawcross D.L., Goldenberg S.D. (2020). Regulation, Risk and Safety of Faecal Microbiota Transplant. Infect. Prev. Pract..

